# DEVICE MEASURED SLEEP QUALITY AND PHYSICAL ACTIVITY IS ASSOCIATED WITH COGNITION IN CHRONIC STROKE

**DOI:** 10.1093/geroni/igae098.3949

**Published:** 2024-12-31

**Authors:** Ryan Stein, Ryan Falck, Daria Tai, Jennifer Davis, Ging-Yuek Hsiung, Janice Eng, Laura Middleton, Teresa Liu-Ambrose

**Affiliations:** University of British Columbia, Vancouver, British Columbia, Canada; University of British Columbia, Vancouver, British Columbia, Canada; University of British Columbia, Vancouver, British Columbia, Canada; The University of British Columbia - Okanagan, Kelowna, British Columbia, Canada; University of British Columbia, Vancouver, British Columbia, Canada; University of British Columbia, Vancouver, British Columbia, Canada; University of Waterloo, Waterloo, Ontario, Canada; University of British Columbia, Vancouver, British Columbia, Canada

## Abstract

Having a stroke increases risk for dementia two-fold. Both moderate to vigorous physical activity (MVPA) and sleep are positively associated with cognitive function. These associations may vary by biological sex. Notably, evidence shows physical activity mitigates the negative impact of poor sleep on cognitive function. Poor sleep quality is a common consequence of stroke and may contribute to cognitive impairment and dementia poststroke. We examined if MVPA attenuates the relationship of device-measured sleep quality and cognitive function in older males and females living with chronic stroke (i.e., > 12 months since stroke). We used baseline data acquired from 97-community-dwelling older adults with chronic stroke (71±9 years; n=38 female) enrolled in a 6-month randomized controlled trial. We measured MVPA and sleep duration using wrist-worn actigraphy. Global cognitive function was measured with the 13-item Alzheimer’s Disease Assessment Scale (ADAS-Cog). We assessed whether MVPA moderated the association between sleep duration and the 13-item ADAS-Cog score in: 1) the full sample; and 2) among males and females separately. Fugl-Meyer score, Montreal Cognitive Assessment score, age, and biological sex were included as covariates. In the full sample, MVPA significantly moderated the association between sleep duration and the 13-item ADAS-Cog score (ß=0.0006±0.0003; p=0.041). Specifically, among individuals with shorter sleep duration, those with greater MVPA had better global cognitive function. This interaction was significant in males (ß=0.0013±0.0005; p=0.016) but not females (ß=0.0004±0.0004; p=0.259). We suggest that MVPA is beneficial for preserving global cognitive function among stroke survivors with shorter sleep duration, particularly among males.

